# Analysis of Methylated Quaternary Ammonium Compounds Using Hydrophilic Interaction Liquid Chromatography Combined With Mass Spectrometry

**DOI:** 10.1002/mas.21942

**Published:** 2025-05-29

**Authors:** Iman Zarei, Ambrin Farizah Babu, Ville M. Koistinen, Marjo Tuomainen, Anna Kårlund, Retu Haikonen, Marko Lehtonen, Kati Hanhineva

**Affiliations:** ^1^ School of Medicine, Institute of Public Health and Clinical Nutrition, Faculty of Health Sciences University of Eastern Finland Kuopio Finland; ^2^ Department of Life Technologies Food Sciences Unit University of Turku Turku Finland; ^3^ School of Pharmacy, Faculty of Health Science University of Eastern Finland Kuopio Finland; ^4^ LC–MS Metabolomics Center, Biocenter Kuopio Kuopio Finland

**Keywords:** acylcarnitines, betainized compounds, electrospray ionization (ESI), hydrophilic interaction liquid chromatography (HILIC), liquid chromatography‐mass spectrometry (LC–MS), metabolomics, methylated quaternary ammonium compound (mQAC)

## Abstract

Liquid chromatography‐mass spectrometry (LC–MS) is a powerful technique for the detection and quantification of methylated quaternary ammonium compounds (mQACs), such as acylcarnitines and methylated amino‐acid‐derived (betainized) compounds, in biological matrices. Due to their high polarity and permanent charge, mQACs present analytical challenges, particularly in achieving efficient chromatographic retention and resolution. Here, we focus on the application of hydrophilic interaction liquid chromatography combined with mass spectrometric (HILIC–MS), for the analysis of these compound classes in biological samples. We highlight practical considerations in their analysis, including their MS/MS fragmentation patterns and identification in positive electrospray mode (ESI)^+^, to support researchers working with mQACs in targeted or untargeted metabolomics studies.

## 1 Introduction

Quaternary ammonium compounds (QACs) are positively charged organic compounds that contain a central nitrogen atom bonded to four clusters of atoms, comprising of alkyl, aryl, or organyl groups (Bureš 2019). The formation of the permanently charged positive cation can be explained by nitrogen having five valence electrons and four sp^3^ hybrid orbitals, of which one is reserved for a lone electron pair. The electron pair gets donated for the fourth covalent bond, the lost electron causing the inherent positive charge (Cox 2004). Methylated quaternary ammonium compounds (mQACs), for example, acylcarnitines and betainized compounds, are a specific subset of QACs, in which one or more of the organic groups attached to the central nitrogen atom are methyl groups (CH_3_). They are widely distributed in nature and can be found in a variety of biological sources, including plants, animals, and microorganisms with diverse biological roles (McCoin et al. 2015; Dambrova et al. 2022; Matern 2022; Killenberger 2023; Koistinen et al. 2019; Kärkkäinen et al. 2018b, 2018b). This similarity in chemical structure may reflect functional similarities between mQACs in biological systems, such as their involvement in cellular signaling and regulation.

*Acylcarnitines* are derivatives of carnitine that are formed through the attachment of an acyl group to the amino group of carnitine. These compounds play a crucial role in the transport of fatty acids into the mitochondria for beta‐oxidation necessary for energy production to maintain cellular activity (Reuter and Evans 2012). Acylcarnitines can be classified based on the carbon atom count in their acyl chains as short‐chain (C_2_–C_5_), medium‐chain (C_6_–C_12_), long‐chain (C_13_–C_20_), and very long‐chain (> C_20_) acylcarnitines and they differ in their prevalence in biological systems as well as association with pathological conditions (Dambrova et al. 2022; van der Hooft et al. 2015). *Betainized compounds* are a group of methylated, typically amino‐acid‐derived compounds. One well‐known example is trimethylglycine also known as glycine betaine or betaine. mQACs (acylcarnitines and betainized compounds) have gained attention for their role in biological applications such as human and animal tissues, due to their significant roles in for example, energy metabolism, lipid transport, and osmoregulation (McCoin et al. 2015; Larsen et al. 2011; Zheng et al. 2021). Therefore, the accurate analysis of these compounds is crucial for studying their functions and roles in various diseases.

In biomedical research, some mQACs serve as potential biomarkers for metabolic diseases. As an example, increased levels of circulating acylcarnitines have been associated with obesity, insulin resistance, and type 2 diabetes (Newgard et al. 2009; Tai et al. 2010; Mihalik et al. 2010), while betainized compounds such as low levels of glycine betaine have been associated with an increased risk of liver diseases (Wang et al. 2021; Chen et al. 2022), and high levels of trimethylamine‐N‐oxide (TMAO), have been associated with an increased risk of cardiovascular diseases (Swanepoel et al. 2022; Wang et al. 2011).

Additionally, in biological systems, glycine betaine functions as an organic osmolyte and a methyl donor in methylation reactions (Burg and Ferraris 2008; Friso et al. 2017; Ueland et al. 2005), thereby playing a vital role in the synthesis of various biomolecules and regulation of many essential cellular processes such as osmoregulation (Larsen et al. 2011; Suprasanna et al. 2016), stress tolerance (Rivera‐Ingraham and Lignot 2017), and membrane stability (Hernandez‐Leon and Valenzuela‐Soto 2023). In addition to glycine betaine, various other, less‐studied betainized compounds have been increasingly reported, especially within the context of nutritional studies (Killenberger 2023; Tuomainen et al. 2019; Landberg et al. 2023). An increased plasma or urinary level of 5‐aminovaleric acid betaine and pipecolic acid betaine has been associated with the intake of whole grains and with improved lipid and glucose metabolism, insulin homeostasis, and inflammation suggesting that the health benefits of whole grains could be partially mediated by the presence of these compounds (Kärkkäinen et al. 2018b; Tuomainen et al. 2019; Pekkinen et al. 2015).

mQACs can be detected using a variety of analytical techniques, including liquid chromatography‐mass spectrometry (LC–MS), gas chromatography‐mass spectrometry (GC–MS), nuclear magnetic resonance spectroscopy (NMR), thin‐layer chromatography, cation‐exchange chromatography, and capillary electrophoresis (Decosterd et al. 2021; MacKinnon et al. 2010; Li‐jun et al. 2002; Preedy 2015). Owing to the chemical properties of mQACs, hydrophilic interaction liquid chromatography combined with mass spectrometry (HILIC–MS) has been proven to be particularly important extension for both targeted, quantitative analytical approaches as well as untargeted metabolite profiling analyses involving these compounds. The technology enables precise profiling of polar compounds such as short‐ and medium‐chain acylcarnitines and betainized compounds across various biological samples with high sensitivity, selectivity and reproducibility (Buszewski and Noga 2012; Cavazzini et al. 2023). Within this context, the current article focuses on benefits and challenges when utilizing HILIC separation‐based LC–MS, for the precise detection, identification and quantification of mQACs such as acylcarnitines and betainized compounds.

## 2 LC–MS Technology for the Detection of mQACs

### 2.1 Chromatographic Separation of mQACs

LC–MS is a commonly applied analytical method for the analysis of mQACs because it allows for the separation and identification of individual species based on their mass‐to‐charge ratios (*m/z*) without the need for complicated sample preparation procedures such as derivatization which is required in gas chromatographic techniques. It also has high sensitivity and dynamic range compared with other techniques, allowing to detect a wide range of concentrations. More specifically, HILIC–MS has shown excellent capabilities in detecting water‐soluble methylated nitrogen compounds (Buszewski and Noga 2012; Cavazzini et al. 2023).

The choice of chromatographic separation techniques is central to the successful detection of mQACs. Among available approaches in LC, HILIC is especially suited for polar and hydrophilic compounds, including mQACs, as it enhances retention and resolution through its hydrophilic stationary phase, which typically consists of materials like nonderivatized silica or silica modified with functional groups, including amide, imide, diol, or zwitterionic groups. These functionalized stationary phases facilitate complex interaction mechanisms, including hydrogen bonding, dipole–dipole interactions, and electrostatic effects, which are essential for resolving highly polar analytes (Cavazzini et al. 2023). When separating mQACs using HILIC, selecting an appropriate stationary phase and optimizing mobile phase conditions are pronounced, especially when working with structurally similar mQACs, such as isomers. The choice of stationary phases, ranging from silica‐based to zwitterionic and amide phases, significantly influences separation efficiency through unique interactions with analytes (Wang et al. 2024).

Amide phases promote hydrogen bonding and dipolar interactions, making them suitable for moderately polar and hydrophilic compounds, including acylcarnitines and betainized molecules. Amide columns can also exhibit mixed‐mode retention mechanisms (Qiao et al. 2018). Amide phases provide stable retention over a broad pH range and are compatible with MS‐friendly buffers (Nováková et al. 2010). Amide columns are highly versatile, as they can be used for both RP and HILIC separations. They offer enhanced selectivity due to the combined effects of hydrophilic and ion‐exchange interactions, allowing for effective separation of complex mixtures, including polar and ionic analytes. Lastly, they are highly efficient and yield good peak shapes, which are essential for accurate and reproducible analytical results (Pesek et al. 2021).

Zwitterionic HILIC phases exhibit both positive and negative charges on the surface, though to a lesser degree than silica and amine‐containing phase, enabling unique selectivity for highly polar and permanently charged compounds (Gilar et al. 2022). These phases often exhibit mixed‐mode retention, combining hydrophilic partitioning with electrostatic interactions (both attraction and repulsion), depending on the analyte's charge and the mobile phase composition. Zwitterionic columns generally perform well across a wide pH range, with enhanced stability and reproducibility at neutral to mildly basic pH (Hu et al. 2024). However, excessive retention and broader peaks may occur for highly charged species unless ionic strength is carefully optimized.

Underivatized silica provides a highly polar surface but is prone to peak tailing and variable retention due to its reactive silanol groups, particularly under low‐buffer or acidic conditions (Carda‐Broch et al. 2018). While it can retain very polar neutral molecules through partitioning, it can be less selective for neutral compounds compared to zwitterionic and amide phases (Gilar et al. 2022).

In addition to the stationary phase, mobile phase composition critically shapes retention and selectivity in HILIC. Acetonitrile (ACN) is commonly used in HILIC due to its ability to enhance retention and selectivity (Kadar et al. 2008). A high initial ACN proportion (typically 70%–95%) is essential to maintain the water‐enriched layer on the stationary phase, which governs partitioning. Decreasing acetonitrile content during a gradient elution enables the differential elution of mQACs based on polarity and hydrophobicity (Kadar et al. 2008).

The buffer type, pH, concentration, and ionic strength in the mobile phase are also critical factors that significantly influence retention and selectivity in chromatographic separations. The type of buffer can substantially alter both retention and selectivity. For instance, formate and acetate buffers may yield different selectivity profiles due to their distinct interactions with the stationary phase (Vikingsson et al. 2008). Additionally, trifluoroacetic acid (TFA) and methanesulfonic acid (MSA) can induce different ion‐exchange behaviors, with TFA favoring anion exchange and MSA promoting cation exchange (McCalley 2017).

The pH of the mobile phase can significantly affect the retention of charged analytes. For example, increasing the pH can increase the retention of anionic compounds while decreasing the retention of cationic compounds (Baškirova et al. 2017). Acidic pH conditions may improve peak shapes for basic compounds by minimizing ion‐exchange interactions (Heaton et al. 2014).

Buffer concentration directly impacts ionic strength and can stabilize the ionized states of analytes and stationary phases, thereby improving reproducibility and retention of charged species (Baškirova et al. 2017; Heaton et al. 2014). It can also influence the structure and stability of the aqueous layer essential for HILIC partitioning (Heaton et al. 2014).

Ionic strength modulates electrostatic interactions. Higher ionic strength may suppress excessive retention of cationic analytes by competing for interaction sites on the stationary phase (Abbood et al. 2017). Additionally, the nature of buffer cations affects retention behavior; for instance, barium‐containing buffers often result in stronger retention than those containing ammonium or lithium cations (Kalíková et al. 2018).

### 2.2 Sensitivity Considerations of mQAC Analysis

mQACs, by nature, possess a permanently positive charge, which makes them particularly well‐suited for detection by ESI. This intrinsic characteristic enhances their ionization efficiency, a critical factor in achieving high sensitivity in mass spectrometry and thus enabling the detection of mQACs even at trace levels (Lupo 2018). In LC–MS, sensitivity is primarily determined by how efficiently analytes in solution are converted into gas‐phase ions (ionization efficiency) and how effectively these ions are transferred from atmospheric pressure into the low‐pressure region of the mass spectrometer (transmission efficiency) (Page et al. 2007). Using a high‐organic mobile phase in HILIC can improve the detection of low‐concentration compounds by providing a higher signal‐to‐noise ratio. This approach also enhances desolvation and ionization in ESI, further contributing to the accurate detection of mQACs. The HILIC–MS method, with its inherent ability to efficiently separate and ionize water‐soluble methylated nitrogen compounds, offers enhanced sensitivity for detecting mQACs (Buszewski and Noga 2012). For example, a quantitative method developed in our laboratory possesses limits of detection for representative compounds such as glycine betaine and carnitine below 10 nM, with linear dynamic ranges spanning up to four orders of magnitude (Tuomainen et al. 2019).

Sensitivity across HILIC–MS methods in the literature can vary substantially based on ion source optimization, stationary phase properties, and sample matrix complexity. Regarding ion source optimization, the design of the ESI source can significantly impact sensitivity (Hsieh et al. 2009). Different mass spectrometers with varying source geometries show different sensitivity gains under HILIC conditions. For example, the 6460 Triple Quadrupole system showed a high sensitivity gain at pH 6 and a flow rate of 300 μL/min, but this gain dropped significantly at higher flow rates and lower pH. Under these conditions, 92% of the tested compounds exhibited an increased signal‐to‐noise ratio compared to RP chromatography (Periat et al. 2014). Conversely, the AB Sciex API 5000 system showed limited sensitivity variation with different flow rates and pH levels (Periat et al. 2014). Additionally, adjusting parameters such as cone and capillary voltages can enhance signal stability and sensitivity, which are highly dependent on solvent composition (Ding et al. 2007; Tycova and Foret 2015; Meikopoulos et al. 2022; Heaton et al. 2019).

The dynamic range of mQACs in biological matrices is extensive. Carnitine and choline may occur at high micromolar concentrations (e.g., 20–60 µm in plasma) (Cao et al. 2011; Xu et al. 2024), while trimethyllysine and butyrobetaine are often present in low nanomolar concentrations (Hirche et al. 2009). Such variability necessitates careful consideration of sample preparation and method linearity to prevent peak saturation or under‐detection. For example, we observed saturation artifacts with glycine betaine in undiluted samples, requiring pre‐analysis dilution to maintain sample concentration within the dynamic range of the method.

Supporting literature confirms that both targeted and untargeted LC–MS approaches offer broad dynamic ranges and excellent sensitivity for QAC‐related compounds. For example, targeted methods for acylcarnitines have achieved limit of quantifications below 0.00018 µg/mL (Zhang et al. 2024), and other approaches report detection of carnitine and acetylcarnitine in serum and urine at concentrations in the nanomolar range, corresponding to approximately 0.00016–0.001 µg/mL (Isaguirre et al. 2013). For betainized compounds, detection limits for betaine, L‐carnitine, and choline ranged from 0.05 to 0.20 µg/mL, with linearity extending up to 100 µg/mL and imprecision below 10% (Wei et al. 2017; Steuer et al. 2016). Untargeted workflows have identified up to 758 acylcarnitines across biological matrices such as plasma, urine, dried blood spots, and tissues (e.g., liver, heart, kidney, and muscle), illustrating the breadth of HILIC–MS in metabolite profiling (Davis et al. 2017; Jung et al. 2016; Gucciardi et al. 2012).

LC–MS quantification of mQACs also demonstrates high analytical precision and accuracy. Intra‐ and inter‐day variability in carnitine quantification are reported to be below 10%, with accuracies ranging from 95.2% to 109.0% (Miller et al. 2012; Vernez et al. 2004). Such reproducibility reinforces the robustness of HILIC–MS approaches for metabolomics and clinical studies. Ion mobility techniques can be employed not only for improved separation but also for enhanced sensitivity by minimizing matrix interference and signal suppression caused by isobaric or co‐eluting compounds (Narváez et al. 2024).

### 2.3 Matrix Interference and Strategies for Signal Optimization in Complex Samples

Matrix interference occurs in both targeted and untargeted LC–MS (Chamberlain et al. 2019; Zhu et al. 2024) and affects several analytical parameters, including selectivity, ionization efficiency, recovery, precision, and accuracy.

Matrix components can co‐elute with analytes, leading to ion suppression or enhancement and thereby compromising the selectivity of LC–MS methods (Nasiri et al. 2021). For example, endogenous phospholipids, especially glycerophosphocholines and lysophosphatidylcholines, are known to co‐elute with acylcarnitines and other mQACs in reversed‐phase LC–MS, resulting in reduced selectivity and poor reproducibility (Xu et al. 2013; Liu et al. 2008; Erngren et al. 2019). In addition, the presence of isobaric or isomeric mQACs, such as structurally similar acylcarnitines or methylated amino acid derivatives, can interfere with the correct identification and quantification of target analytes (Yan et al. 2008).

Matrix effects such as ion suppression or enhancement directly impact the ionization efficiency of mQACs, especially under ESI conditions. The degree of ion suppression is influenced by both the physicochemical properties of the analytes (e.g., polarity, charge) (Omari et al. 2019), the complexity of the matrix (Rossmann et al. 2015), and the ion source construction (Page et al. 2007).

Sample preparation plays a crucial role in mitigating matrix interference. Techniques such as liquid–liquid extraction (LLE), and solid‐phase extraction (SPE) have been applied to improve recovery of mQACs (Ghosh et al. 2011; Liu and Aubry 2013; Palandra et al. 2007). However, even with optimized protocols, extraction efficiency can vary considerably depending on the matrix. For instance, fecal samples rich in fiber, bile acids, and microbial metabolites can trap or chemically interact with polar mQACs, leading to inconsistent recovery and elevated matrix background (Monteil‐Rivera et al. 2024). Therefore, method development must consider matrix‐specific characteristics and optimize extraction accordingly.

Matrix interferences often lead to signal distortion, which undermines the accuracy and precision of quantitative LC–MS results (Hewavitharana 2021). This is especially problematic in targeted metabolomics workflows, where consistent quantification across sample types is essential (Jia et al. 2023). The use of stable isotope‐labeled internal standards (SIL‐IS) is considered the gold standard to correct for matrix effects and improve quantitation reliability (Radovanovic et al. 2022). SIL‐IS compensate for both extraction losses and ion suppression, provided they co‐elute with the analyte and share similar chemical behavior.

Given this, matrix interferences pose a substantial challenge in both targeted and untargeted metabolomics workflows, but various strategies, including enhanced sample extraction, the use of SIL‐IS and more advanced techniques, such as isotope dilution, can help mitigate these effects and improve quantification accuracy.

### 2.4 An Example Method for the HILIC–MS Analysis of mQACs

In our laboratory, we have employed the same HILIC method to analyze mQACs in both untargeted metabolite profiling and in quantitative targeted analyses with a triple quadrupole instrument (Table 1) (Kärkkäinen et al. 2018a; Tuomainen et al. 2019; Babu et al. 2022). For example, acylcarnitines, ranging from short‐chain (C_2_‐C_6_) to very long‐chain (> C_20_) compounds (Meikopoulos et al. 2022; Babu et al. 2022; Yu et al. 2018), have been effectively separated using an amide‐based HILIC column. Retention characteristics depend on both chain length and degree of saturation; for instance, short‐chain acylcarnitines can be effectively separated with a mobile phase consisting of 80:20 acetonitrile‐water, buffered with 10 mM ammonium acetate at pH 4.0, targeting their polar head groups. In contrast, although detectable with HILIC, longer hydrophobic acylcarnitines are better suited for separation by RP chromatography (Klåvus et al. 2020).

**Table 1:** Chromatographic conditions used for both nontargeted metabolomics and quantitative analyses of methylated quaternary ammonium compounds (mQACs) (Kärkkäinen et al. 2018a; Tuomainen et al. 2019; Klåvus et al. 2020).

Parameter	Conditions
Column	AcQuity UPLC BEH Amide, 1.7 μm, 2.1 × 100 mm (Waters)
Column temperature	45°C
Mobile phases	(A) 50% ACN in H₂O, including 20 mM ammonium formate, 0.25% formic acid
	(B) 90% ACN in H₂O, including 20 mM ammonium formate, 0.25% formic acid
Elution gradient	0 min → 2.5 min, 100% B; 2.5 min → 10 min, 100% B → 0% B; 10 min → 10.01 min, 0% B → 100% B; 10.01 min → 12.5 min, 100% B
Flow rate	0.6 mL/min
Sample tray T	4 or 10°C
Injection volume	1–2 μL

We also demonstrated that betainized compounds are well‐suited for separation on HILIC columns with amide phases. For instance, a mobile phase of 70% acetonitrile and 30% water, buffered with 10 mM ammonium formate at pH 3.0, has provided optimal separation in our experiments. This buffer helps manage the strong ionic interactions characteristic of these compounds, thus enhancing both detection sensitivity and selectivity. In terms of untargeted MS analysis, the key parameters include a drying gas temperature of 325°C and a sheath gas temperature of 350°C, with a flow rate of 10 L/min (Tuomainen et al. 2019). For quantitative analyses, the Triple Quadrupole LC–MS system with an electrospray ion source has been used, offering high precision via multiple reaction monitoring (MRM) (Tuomainen et al. 2019).

## 3 Identification of mQACs

**Figure 1:**
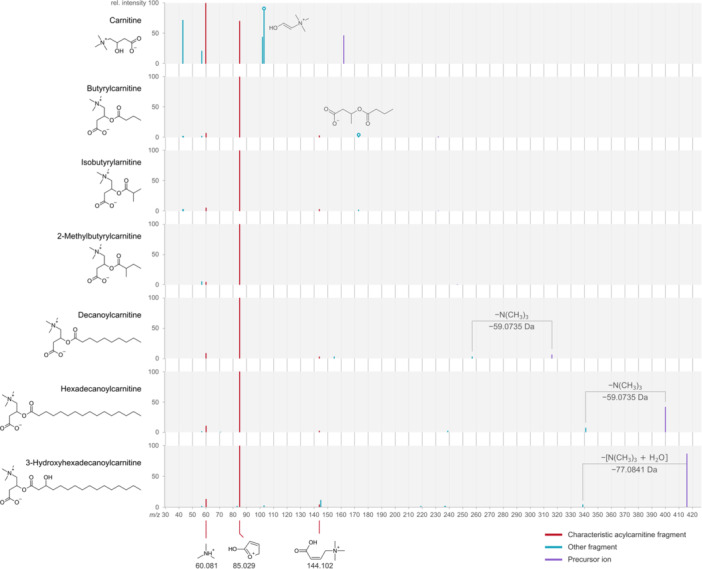
Schematic representation of carnitine and acylcarnitine fragmentation in LC–MS, HILIC–MS at +20 eV collision energy using collision‐induced dissociation (CID). The typical neutral losses and the tentative structures of the characteristic acylcarnitine fragments are presented.

Isomerism is a key challenge in the structural elucidation of mQACs and can occur due to structural isomerism (such as the presence of branched chains or side‐chain hydroxyl/methyl groups), *cis*–*trans* isomerism (such as double bonds in the acyl chains of acylcarnitines), and stereoisomerism (such as the d and l isomers of carnitine and some amino‐acid‐derived betaines). The identification of these compound classes and their isomers necessitates efficient chromatographic separation and careful interpretation of the product ion spectrums. Thus, in this section, we have explored the importance of fragmentation patterns in the identification of acylcarnitines and betainized compounds. We have also shown some examples of isomers (e.g., butyrylcarnitine and isobutyrylcarnitine from Figure 1, and 5‐aminovaleric acid betaine, valine betaine, 3‐methyl‐4‐(trimethyl‐ammonio)butanoic acid, and 4‐aminovaleric acid betaine from Table 3) to highlight the challenge of isomerism and the use of HILIC to overcome it.

### 3.1 Fragmentation Pattern of Acylcarnitines

The fragmentation pattern of acylcarnitines in product ion spectra measured with ESI^+^ depends on the structure and length of the acyl chain (Chen et al. 2015), the collision energy (Tu and Muddiman 2019), and instrument parameters (Simões et al. 2008). While mobile phase composition and sample matrix do not directly alter fragmentation pathways, they can influence adduct formation and ionization efficiency, which in turn affect the precursor ions selected for fragmentation and the resulting spectral profiles (Chen et al. 2015; Tu and Muddiman 2019; Simões et al. 2008; Sándor et al. 2018). For instance, variations in the acyl chain saturation or branching can lead to distinct fragment ions and relative ion abundances (Chen et al. 2015). Higher collision energies typically increase the extent of fragmentation, altering the spectral appearance (Tu and Muddiman 2019), while instrument‐specific factors, such as ion trap versus Q‐TOF architectures, may influence both the fragmentation pathway and the stability of labile intermediates like noncovalent dimers (Simões et al. 2008). Furthermore, mobile phase composition affects adduct formation and fragmentation behavior. For instance, the inclusion of volatile buffers and additives such as ammonium formate or acetate or triethylamine can promote the formation of ammonium adducts, thereby enhancing ionization efficiency and affecting fragmentation pathways. The type of adduct formed (e.g., sodium, ammonium) can shift the dominant fragmentation routes and alter ion intensities in MS analysis (Chen et al. 2015; Simões et al. 2008; Sándor et al. 2018). In contrast, basic modifiers like triethylamine are typically avoided in LC–MS workflows due to their tendency to suppress ionization and contaminate the mass spectrometer source (Goyon and Zhang 2020).

In addition to MS/MS fragmentation, retention time also plays an important role in the identification process, particularly when dealing with isobaric or isomeric species that produce nearly identical spectra. The retention time is influenced by molecular features such as length of the carbon chain, number of double bonds, and polarity (e.g., hydroxylated, branched, dicarboxyl group), which affect interactions with the stationary phase.

The patterns for acylcarnitine fragmentation are based on characteristic fragments from both the fragments from carnitine and acyl moieties, as well as neutral loss peaks from precursors (Figure 1, Table 2). In the ESI^+^ mode, all types of acylcarnitine species produce a similar product ion with a *m/z* of 85, which is formed through the cleaving of the hydroxybutyrate moiety of carnitine (C_4_H_5_O_2_^+^, *m/z* 85.0290). Another common fragment resulting from the decomposition of trimethylamine (C_3_H_10_N^+^) at *m/z* 60.0813 is also observed. Notably, trimethylamine fragment is also observed in betainized compounds. Generally, the hydroxybutyrate moiety of carnitine (C_4_H_5_O_2_^+^) fragment is the most prominent peak in acylcarnitine product ion spectra. It consistently exhibits high abundance under commonly used collision energies (Yan et al. 2020; Minkler et al. 2008). However, at higher collision energies, the intensity of the fragment corresponding to the hydroxybutyrate moiety of carnitine may decrease. Carnitine easily loss water and produce ([C_7_H_14_NO_2_]^+^) fragment (*m/z* 144.194) is another prominent fragment that can also be used to identify acylcarnitines. These compounds can further be identified by the neutral loss of the precursor ions including trimethylamine (C_3_H_9_N, 59.0735 Da), CH_2_O_2_ (46.0055 Da), C_2_H_4_O_2_ (60.0211 Da), and C_7_H_13_NO_2_ (143.0946 Da). Especially, the neutral loss of the trimethylamine fragment is a useful indicator for identifying co‐eluting nonacylcarnitine compounds. Other fragments resulting from the decomposition of the acyl moiety such as losses of CO, H_2_O, C_2_H_4_, and C_2_H_2_O may also aid in the identification of the acylcarnitine species. These rules (i.e., prominent fragments, fragmentation patterns, and specific neutral losses) can be used to identify and quantify acylcarnitines with even‐ and odd‐numbered carbon atoms, as well as isomeric forms of acylcarnitines. They can also be used to screen for yet unknown acylcarnitine species. However, due to the aforementioned similarity in the fragmentation pattern among the acylcarnitines, chromatographic separation is crucial when dealing with isobaric acylcarnitines or those that occur as positional isomers.

**Table 2:** Major MS/MS fragments and neutral losses of acylcarnitines in ESI^+^ ionization.

Identifier type	Identification	Mass‐to‐charge ratio (*m/z*)	Significance
Fragment	Hydroxybutyrate moiety of carnitine (C_4_H_5_O_2_^+^)	85.0289	Prominent peak in spectra
Fragment	Trimethylamine (C_3_H_10_N^+^)	60.0813	Characteristic fragment of both acylcarnitines and betainized compounds
Fragment	Carnitine − H_2_O (C_7_H_14_NO_2_^+^)	144.1025	Minor characteristic peak
Neutral loss	Trimethylamine (C_3_H_9_N^+^)	59.0735	Important for identifying compounds, structure‐dependent

### 3.2 Fragmentation Pattern of Betainized Compounds

In the context of mass spectrometry, the qualitative analysis of betainized compounds requires a comprehensive understanding of their fragmentation patterns. This section explores the product ion spectra of betainized compounds and their implications for HILIC–MS methods. This is important to note that betainized compounds do not exhibit a unified fragmentation pattern within the compound class. However, the majority of them but not all, share a common fragment corresponding to the loss of trimethylamine (C_3_H_10_N^+^) at *m/z* 60.0813, a pattern also observed in acylcarnitines. In addition to this class‐characteristic fragment, many betainized compounds are structurally derived from amino acids, and their fragmentation spectra can reflect this origin. Specifically, some betainized compounds retain diagnostic fragments that correspond to the backbone or side‐chain moieties of their parent amino acids. Recognizing these fragments can be especially valuable for structure elucidation and annotation of unknowns in complex biological samples. For example, alanine betaine and glutamine betaine show fragments that correspond to the characteristic fragments of alanine and glutamine, respectively (Table 3). Therefore, we included the corresponding amino acids to aid in the interpretation of compound‐specific fragmentation and to support the identification of betainized compounds with similar origins or structures.

**Table 3:** The common spectral fragments of betainized compounds and their corresponding amino acids in ESI^+^.

Betainized compound	*m/z* [M + H]^+^	Corresponding amino acid	*m/z* [M + H]^+^	Common fragment(s) (*m/z*)^a^
(beta‐)Alanine betaine (Trimethyl(beta‐)alanine) (C_6_H_13_NO_2_)	132.1025	Alanine (C_3_H_7_NO_2_)	90.0555	44.05
(Glutamine) betaine (*N*,*N*,*N*‐trimethylglutamine) (C_8_H_16_N_2_O_3_)	189.1239	Glutamine (C_5_H_10_N_2_O_3_)	147.0770	84.04
Trimethyllysine (C_9_H_20_N_2_O_2_)	189.1603	Lysine (C_6_H_14_N_2_O_2_)	147.1134	41.05 56.05
Valine betaine (C_8_H_18_NO_2_)	161.1416	Valine (C_5_H_11_NO_2_)	118.0868	55.05 59.05
Histidine trimethylbetaine (C_9_H_15_N_3_O_2_)	198.1243	Histidine (C_6_H_9_N_3_O_2_)	156.0773	68.05 95.06
Glutamic acid betaine (C_8_H_15_NO_4_)	189.1001	Glutamic acid (C_5_H_9_NO_4_)	148.0610	84.08
Isoleucine betaine (Trimethylisoleucine) (C_9_H_19_NO_2_)	174.1494	Isoleucine (C_6_H_13_NO_2_)	132.1025	58.06 73.06
Phenylalanine betaine (C_12_H_17_NO_2_)	208.1338	Phenylalanine (C_9_H_11_NO_2_)	166.0868	79.05 93.07 103.05 107.05
Proline betaine (C_7_H_13_NO_2_)	144.1025	Proline (C_5_H_9_NO_2_)	116.0712	58.06 70.06

a Based on +20 eV collision energy.

On the other hand, HILIC–MS can be useful for the analysis of isomeric betainized compounds with the same molecular formula but different structures and properties. One example of structural isomeric betainized compounds that can be separated and identified by HILIC–MS and reported by Haikonen et al. (2022). includes 5‐aminovaleric acid betaine (5‐AVAB), 4‐aminovaleric acid betaine (4‐AVAB), and 3‐methyl‐4‐trimethylammoniobutanoate (3M‐4‐TMAB). These compounds were separated using an amide‐based HILIC column, and their elution order was as follows: 5‐AVAB eluted first, followed by 3M‐4‐TMAB, and finally 4‐AVAB. Detailed retention times and exact *m/z* values for these metabolites are provided in Table 4.

**Table 4:** Examples of betainized compounds with their synonyms, Human Metabolome Database (HMDB) (Wishart et al. 2022) and Chemical Entities of Biological Interest (ChEBI) (Hastings et al. 2016) identifiers, molecular formula, retention time, theoretical mass‐to‐charge ratio (m/z), and characteristic MS/MS fragments.

Name	Synonyms and abbreviations	HMDB ID	ChEBI	Molecular formula	RT (Klåvus et al. 2020)^a^	*m/z* [M + H]^b^	Characteristic fragments, +20 eV
Trimethylamine *N*‐oxide	TMAO	HMDB0000925	15724	C_3_H_9_NO	1.48	76.0762	58.065 (100), 59.073 (60)
Glycine betaine	Betaine, trimethylglycine	HMDB0000043	17750	C_5_H_11_NO_2_	3.53	118.0868	59.074 (100), 58.066 (92)
(beta‐)Alanine betaine		HMDB0240479	145235	C_6_H_13_NO_2_	3.74	132.1025	59.074 (100), 58.066 (79), 132.102 (25)
Trigonelline	*N*‐Methylnicotinic acid	HMDB0000875	18123	C_7_H_7_NO_2_	4.03	138.0555	92.050 (100), 94.065 (87), 78.034 (43), 65.038 (28), 93.057 (16)
Proline betaine	Stachydrine	HMDB0004827	35280	C_7_H_13_NO_2_	3.46	144.1025	144.102 (100), 58.066 (45), 84.081 (32)
4‐Trimethylammonio‐butanoic acid	4‐Aminobutyric acid betaine, deoxycarnitine, *gamma*‐butyrobetaine	HMDB0001161	1941	C_7_H_15_NO_2_	3.36	146.1181	43.019 (100), 87.045 (76), 45.034 (42), 60.081 (23), 58.066 (20)
Homostachydrine	Pipecolic acid betaine	HMDB0033433	5757	C_8_H_15_NO_2_	2.86	158.1181	58.065 (100), 158.118 (33), 70.065 (29), 98.095 (26), 56.049 (19), 44.049 (14), 43.041 (14), 42.034 (10)
Betonicine	Hydroxyproline betaine	HMDB0029412	81	C_7_H_13_NO_3_	4.46	160.0974	58.064 (100), 70.065 (35), 42.034 (24), 82.065 (22)
5‐Aminovaleric acid betaine	5‐AVAB; delta‐valerobetaine	HMDB0240732	145234	C_8_H_17_NO_2_	1.94	160.1338	55.055 (100), 60.081 (92), 101.059 (49), 83.049 (33), 43.018 (22), 59.050 (19), 160.133 (12)
Valine betaine		HMDB0240571	n/a	C_8_H_17_NO_2_	2.13	160.1338	60.081 (100), 59.073 (55), 58.066 (51), 160.133 (29)
3‐Methyl‐4‐(trimethylammonio)butanoic acid	3M‐4‐TMAB	n/a†	n/a	C_8_H_17_NO_2_	2.54	160.1338	55.054 (100), 59.049 (92), 60.081 (85), 101.059 (45), 83.050 (30), 160.134 (10)
4‐Aminovaleric acid betaine	4‐AVAB	n/a	145241	C_8_H_17_NO_2_	3.03	160.1338	55.054 (100), 101.059 (62), 60.081 (60), 83.049 (34), 59.049 (25), 43.018 (9)
Carnitine		HMDB0000062	17126	C_7_H_15_NO_3_	4.60	162.1130	60.081 (100), 103.039 (91), 43.018 (72), 85.029 (70), 162.112 (46), 102.092 (44), 57.034 (21)
*N*6,*N*6,*N*6‐Trimehyllysine		HMDB0001325	176482	C_9_H_20_N_2_O_2_	6.80	189.1603	84.081 (100), 60.081 (17), 130.086 (12)
Phenylalanine betaine		HMDB0240552	90138	C_12_H_17_NO_2_	1.44	208.1338	103.054 (100), 107.049 (93), 60.081 (59), 208.137 (37), 131.049 (36), 79.054 (31)
Lenticin	Tryptophan betaine	HMDB0061115	5832	C_14_H_18_N_2_O_2_	1.30	247.1447	146.061 (100), 188.072 (57), 60.082 (50), 118.066 (19), 144.082 (18)

a RT value acquired with the method described in Klåvus et al. (Klåvus et al. 2020).

^b^ PubChem CID: 20606311.

**Figure 2:**
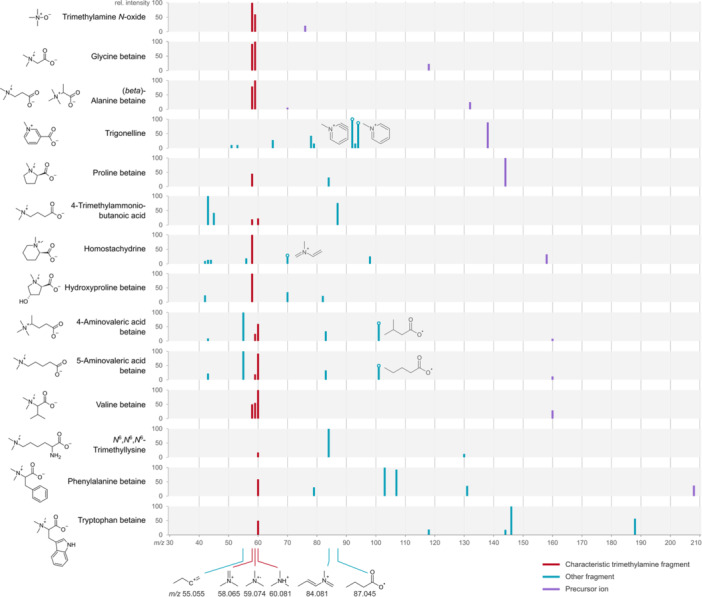
Schematic representation of the fragmentation of betainized compounds in LC–MS, HILIC–MS at +20 eV collision energy except for homostachydrine and hydroxyproline betaine, which are at +40 eV. The tentative structure of the characteristic and some other key fragments is presented. Data from Koistinen (Koistinen 2019).

These three isomeric betainized compounds have the same *m/z* of 160 in positive ion mode and share many common product ions in MS/MS spectra. Therefore, MS alone cannot reliably distinguish these compounds. However, HILIC enables chromatographic separation based on differences in polarity and interaction with the stationary phase, thereby allowing resolution of these isomers based on their retention times. By coupling HILIC with MS, more reliable and confident identification of such isomeric compounds becomes possible. Figure 2 (Koistinen 2019) and Table 4 (Klåvus et al. 2020) provide examples of betainized compounds, their associated fragmentation pattern, and main spectra, all based on pure standards.

## 4 Conclusion

The HILIC–MS method is a powerful technique for the analysis of water‐soluble mQACs, providing improved separation, sensitivity, and detection capabilities, thereby facilitating research and applications in biological research. These advancements have expanded our understanding of the occurrence, fate, and potential impacts of mQACs, and future improvements in LC–MS instrumentation and method development, including the integration enhanced sample preparation techniques, labeled internal standards, will further enhance the analysis of mQACs.

## Author Contributions

**Kati Hanhineva:** conceptualization, writing – review and editing, supervision. **Iman Zarei:** conceptualization, writing – original draft preparation, writing – review and editing, table processing. Ambrin **Farizah Babu:** writing – original draft preparation, writing – review and editing. **Ville M. Koistinen:** writing – review and editing, visualization, table processing. **Marjo Tuomainen:** writing – review and editing, table processing. **Anna Kårlund:** writing – review and editing. **Retu Haikonen:** writing – review and editing. marko lehtonen: writing – review and editing. All authors critically revised the manuscript.

## Conflicts of Interest

V.M.K., A.F.B., and K.H. are affiliated with Afekta Technologies Ltd. The other authors declare no conflicts of interest.
